# The Role of Anesthetic Management in Lung Cancer Recurrence and Metastasis: A Comprehensive Review

**DOI:** 10.3390/jcm13226681

**Published:** 2024-11-07

**Authors:** Jaewon Huh, Wonjung Hwang

**Affiliations:** Department of Anesthesiology and Pain Medicine, Seoul St. Mary’s Hospital, College of Medicine, The Catholic University of Korea, Seoul 06591, Republic of Korea; ether@catholic.ac.kr

**Keywords:** anesthesia, cancer recurrence, lung cancer, metastasis, perioperative care

## Abstract

Lung cancer remains a leading cause of cancer-related mortality worldwide. Although surgical treatment is a primary approach, residual cancer cells and surgery-induced pathophysiological changes may promote cancer recurrence and metastasis. Anesthetic agents and techniques have recently been shown to potentially impact these processes by modulating surgical stress responses, immune function, inflammatory pathways, and the tumor microenvironment. Anesthetics can influence immune-modulating cytokines, induce pro-inflammatory factors such as HIF-1α, and alter natural-killer cell activity, affecting cancer cell survival and spread. Preclinical studies suggest volatile anesthetics may promote tumor progression by triggering pro-inflammatory signaling, while propofol shows potential antitumor properties through immune-preserving effects and reductions in IL-6 and other inflammatory markers. Additionally, opioids are known to suppress immune responses and stimulate pathways that may support cancer cell proliferation, whereas regional anesthesia may reduce these risks by decreasing the need for systemic opioids and volatile agents. Despite these findings, clinical data remain inconclusive, with studies showing mixed outcomes across patient populations. Current clinical trials, including comparisons of volatile agents with propofol-based total intravenous anesthesia, aim to provide clarity but highlight the need for further investigation. Large-scale, well-designed studies are essential to validate the true impact of anesthetic choice on cancer recurrence and to optimize perioperative strategies that support long-term oncologic outcomes for lung cancer patients.

## 1. Introduction

Lung cancer remains one of the most prevalent cancers with a high mortality rate [1]. According to 2020 data, it is the most frequently diagnosed cancer worldwide, accounting for 11.4% of all cancer cases and causing approximately 1.8 million deaths annually. Non-small cell lung cancer (NSCLC), which comprises about 85% of all lung cancer cases, is primarily treated with surgical resection. However, despite the successful removal of the primary tumor, microscopic residual cancer cells often persist, leading to recurrence and metastasis. Postoperative recurrence and metastasis rates in NSCLC patients range from 30% to 55%, with a median survival time of approximately 21 months.

Ideally, the immune system would eliminate residual cancer cells after surgery [2]. However, surgical interventions often exacerbate pathophysiological changes that hinder this process. The surgical stress response plays a crucial role in disrupting inflammatory balance, resulting in immunosuppression [3,4]. Additionally, the preoperative period, characterized by patient anxiety and stress, can elevate cortisol and catecholamine levels, leading to reduced natural-killer (NK) cell activity and weakened immune readiness. The postoperative period also poses challenges, such as pain, surgical site inflammation, and potential infections, which further compromise immune recovery and promote an environment conducive to tumor growth. These stressors collectively contribute to inflammatory and immune system alterations that affect cancer outcomes.

The anesthesiologist’s role extends beyond the intraoperative period to include managing these preoperative and postoperative stress responses. Techniques such as preemptive analgesia, multimodal pain management, and the targeted use of sedatives can mitigate these stress-induced effects. Through these strategies, anesthesiologists play a vital role in maintaining immune function and reducing the risk of cancer recurrence. Thus, understanding these mechanisms is essential for developing strategies to mitigate surgery-induced effects that contribute to cancer progression.

Emerging evidence suggests that anesthetic agents and techniques may also influence cancer progression. This review aims to provide an in-depth analysis of the mechanisms underlying cancer recurrence and metastasis following surgery. Additionally, we will explore perioperative strategies and their role in mitigating cancer recurrence risk, particularly focusing on lung cancer patients.

## 2. Materials and Methods

A comprehensive literature search was conducted using electronic databases, including PubMed, EMBASE, Web of Science, Google Scholar, and the Cochrane Library. The search utilized the following keywords: “cancer recurrence”, “metastasis”, “anesthesia”, “analgesia”, “anesthetic agent”, and “lung cancer”. Studies published in English up to December 2023 were included, and there were no restrictions on study type, ensuring a broad and inclusive scope for eligible studies.

**Figure 1:**
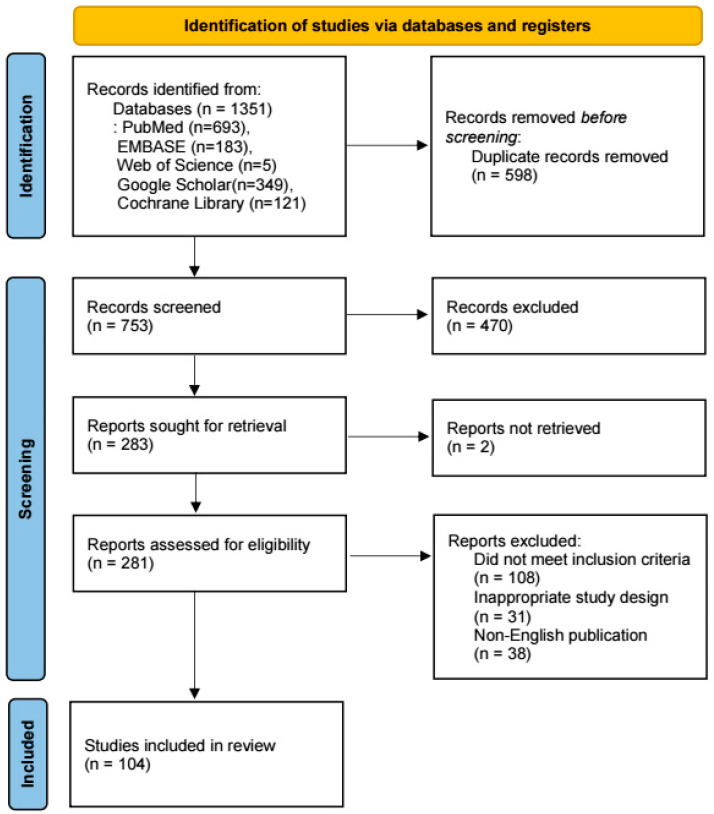
PRISMA flow diagram.

We adhered to the PRISMA (Preferred Reporting Items for Systematic Reviews and Meta-Analyses) guidelines in identifying, screening, and selecting studies (Figure 1). All retrieved articles were manually examined, and additional studies were identified by screening the reference lists of relevant reviews and articles. The selection criteria included both preclinical and clinical studies examining the role of anesthetic agents and techniques in cancer recurrence and metastasis, with a specific focus on lung cancer surgery. Studies investigating the effects of general and regional anesthesia, volatile anesthetics, opioids, and non-opioid agents were prioritized.

## 3. Mechanisms of Cancer Recurrence After Surgery

### 3.1. Remnants of Cancer Cells and Circulating Tumor Cells

Despite curative surgical resection being the primary treatment for solid tumors, microscopic residual cancer cells often persist, leading to local recurrence, lymphatic or vascular invasion, and transcoelomic dissemination, such as intrapleural or intraperitoneal spread [5]. Circulating tumor cells (CTCs) play a critical role in distant metastasis, as they can escape the primary tumor site and travel through the bloodstream [6]. CTCs are frequently detected in patients with solid tumors, and several studies have shown elevated CTC levels following surgery for cancers such as lung, hepatocellular, gastric, colorectal, and breast [7,8,9,10,11]. Elevated CTC counts are generally associated with a poor prognosis; however, not all CTCs lead to metastasis. For metastasis to occur, CTCs must evade immune surveillance, survive in the circulatory system, and successfully colonize distant organs. This process is facilitated by postoperative stress responses, inflammation, and immunosuppression, which collectively create an environment favorable for tumor cell survival and progression. The ability of CTCs to evade immune destruction and establish secondary tumors is significantly influenced by perioperative disruptions in immune and inflammatory pathways.

### 3.2. Tumor Microenvironment and Metastasis

Cancer cells reside within a tumor microenvironment (TME), composed of various elements, including inflammatory and immune cells, stromal cells, blood vessels, and extracellular matrix components [12,13]. Surgical manipulation and perioperative stress response can significantly disrupt the TME, triggering a cascade of events that facilitate cancer cell migration to distant sites [12,14]. First, cancer cells acquire invasive and migratory properties through epithelial–mesenchymal transition (EMT), during which they transform into fibroblast-like cells. Second, the transformed cancer cells infiltrate adjacent tissues, eventually entering the circulation by penetrating lymphatic or blood vessels. During this phase, CTCs may be recognized and targeted by immune surveillance mechanisms, such as NK cells or cytotoxic T (Tc) cells. Third, surviving CTCs travel to distant sites and function as progenitor cells. Finally, these progenitor cells interact with local tissue, inflammatory cells, and other components to proliferate within the newly formed TME.

The complex and dynamic interactions between cancer cells and surrounding non-malignant cells within the TME are pivotal in cancer progression and metastasis. Inflammatory cells, for instance, contribute to cancer invasion and proliferation by releasing cytokines, chemokines, growth factors, and enzymes [15,16]. Cytokines and chemokines produced by inflammatory cells attract and activate immune cells while also promoting cancer cell migration and invasion. Growth factors, such as epidermal growth factor (EGF) and vascular endothelial growth factor (VEGF), stimulate cancer cell proliferation, survival, and angiogenesis. Additionally, enzymes such as matrix metalloproteinases (MMPs) degrade the extracellular matrix at the invasive front, facilitating cancer cell invasion into surrounding tissues.

### 3.3. Surgery-Induced Pathophysiologic Changes and Cancer Recurrence

**Figure 2:**
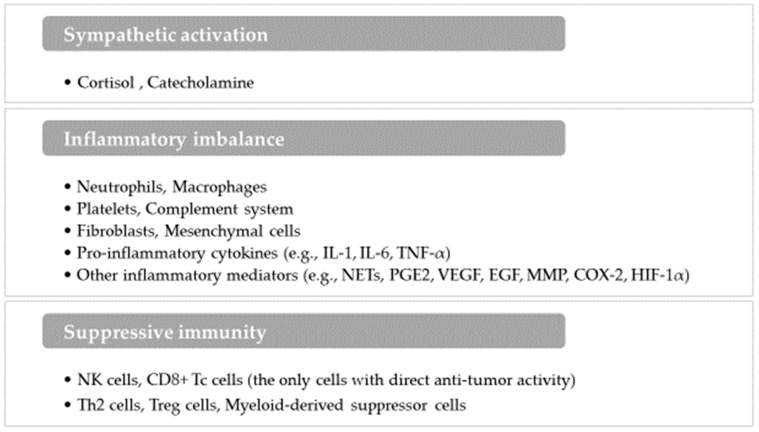
Overview of tumor-promoting mechanisms during surgical treatment. The diagram illustrates the key mechanisms and related factors influencing cancer progression during the perioperative period. Notably, NK cells and CD8+ Tc cells are indicated as the only immune elements providing direct anti-tumor activity, contrasting with other factors that promote tumor growth. IL: interleukin, TNF-α: tumor necrosis factor—alpha, NETs: neutrophil extracellular traps, PGE2: prostaglandin E2, VEGF: vascular endothelial growth factor, EGF: epidermal growth factor, MMP: matrix metalloproteinase, COX-2: cyclooxygenase-2, HIF-1α: hypoxia-inducible factor-1 alpha, NK cells: natural killer cells, CD8+ Tc cells: CD8+ cytotoxic T cells, Th2 cells: helper T2 cells, Treg cells: regulatory T cells.

Surgical stress is induced not only by tissue trauma but also by several factors such as hypothermia, tissue hypoxia, transfusion, and patient anxiety. These stressors initiate a cascade of sympathetic, inflammatory, and immune system changes, each of which can influence the metastatic process [4,17] (Figure 2).

#### 3.3.1. Sympathetic Activation

Surgical stress primarily activates the sympathetic nervous system, resulting in an increased secretion of cortisol and catecholamines. These neuroendocrine mediators elevate inflammatory cytokines (e.g., IL-6, IL-8) and immunosuppressive cytokines (e.g., IL-4, IL-10, VEGF), suppressing NK cell and Tc cell activity while promoting regulatory T (Treg) cell expansion, ultimately contributing to tumor progression [5].

Catecholamine can directly bind to β-receptors on tumor cells, inducing morphological changes that promote EMT [18]. Additionally, it can indirectly contribute to the remodeling of the TME by stimulating the secretion of IL-6 (an inflammatory cytokine), VEGF (a proangiogenic factor), and MMP-2/9 (enzymes involved in extracellular matrix degradation). The activation of β-receptors on the surface of cancer cells has been shown to accelerate metastasis and tumor growth in breast, colon, liver, prostate, and lung cancers [19,20].

#### 3.3.2. Inflammatory Imbalance

Surgical tissue damage and sympathetic stimulation trigger an inflammatory response as part of the normal wound-healing process [21]. The acute inflammatory response is primarily mediated by macrophages and neutrophils, which secrete pro-inflammatory cytokines such as IL-1, IL-6, and TNF-α. This response initially promotes a helper T (Th)1-dominant profile, essential for cell-mediated immunity through the secretion of interferon gamma (IFN-γ) and IL-2. However, persistent inflammatory cell stimulation results in excessive cytokine production, altering the Th1/Th2 ratio and leading to an inflammatory imbalance [22,23]. This suppresses the activity of NK cells and CD8+ Tc cells while enhancing the functions of Th2 cells and Treg cells, thereby weakening anti-tumor immunity and facilitating tumor progression. Additionally, fibroblasts and mesenchymal cells secrete several factors, including growth factors (e.g., VEGF, EGF), enzymes (e.g., MMP, COX-2), transcription factors (e.g., HIF-1α, NF-kB, STAT-3), and chemokines (e.g., CXCR-2). These molecules are pivotal in tumor growth, angiogenesis, and consequent dissemination.

IL-6 stimulates macrophages to secrete prostaglandin E2 (PGE2), further amplifying the inflammatory response and inhibiting cell-mediated immunity. PGE2 also enhances tumor cell migration and angiogenesis, facilitating metastasis [24,25]. In lung cancer models, PGE2 has been shown to upregulate MMP-9 mRNA expression while downregulating E-cadherin mRNA expression [26]. These changes enhance extracellular matrix degradation and reduce cell adhesion, promoting cancer cell invasion and metastasis.

Neutrophils also contribute to cancer progression and dissemination by releasing neutrophil extracellular traps (NETs) [27]. While NETs play an essential role in clearing microorganisms, they promote tumor cell proliferation, migration, and invasion in the context of cancer. In addition, NETs interact with CTCs, facilitating their implantation in distant tissues and promoting metastasis. [28]. These processes are mediated by releasing high mobility group box 1 (HMGB1) and activating Toll-like receptor (TLR) 9-dependent pathways.

Platelets play a dual role in their interaction with CTCs. First, they can form platelet-CTC aggregates, shielding CTCs from immune surveillance [29]. Second, activated platelets release factors such as TGF-β, platelet-derived growth factor (PDGF), and ATP, which further modulate the TME to favor cancer growth [30]. TGF-β suppresses NK cell activity and other immune responses, creating an immunosuppressive environment, while PDGF promotes tumor growth and angiogenesis. Furthermore, ATP enhances vascular permeability, facilitating the infiltration of immune cells and other factors into the TME. Perioperative increases in platelet levels have been linked to poor cancer prognosis [31].

Recent studies have highlighted the role of fibrinogen and the complement system in enhancing the metastatic process. Surgery-induced pro-inflammatory cytokines elevate fibrinogen levels, forming fibrin complexes around tumor cells that protect them from NK cell surveillance and promote tumor adhesion to endothelial cells [32,33]. The complement system is also activated during surgery, contributing to cancer recurrence by promoting cancer cell stemness, enhancing angiogenesis, and inhibiting anti-tumor immunity [34,35,36,37]. In lung cancer, complement activation through the C3a receptor has been shown to promote tumor progression by influencing T cell differentiation and fostering an immunosuppressive microenvironment [38].

Tissue hypoxia, a common consequence of surgery, induces the expression of hypoxia-inducible factor (HIF)-1α, which promotes angiogenesis by upregulating VEGF [39,40]. This pathway not only aids tissue repair but also provides cancer cells with a route for distant metastasis. The overexpression of HIF-1α and VEGF has been associated with poor prognosis in various cancer types [41,42].

#### 3.3.3. Suppressive Immunity

Perioperative stress and inflammatory imbalances can impair the body’s anti-tumor immune response, reducing its ability to eliminate residual cancer cells after tumor resection [43]. The peak suppression of immune function typically occurs around the third day after surgery, with full recovery taking up to two weeks [44]. During this period, cancer cells may evade immune detection and establish a tumor-promoting microenvironment conducive to metastasis [45]. Tumor cells can express surface ligands that inhibit NK cell cytotoxicity, allowing them to evade immunosurveillance. Additionally, tumor cells release inflammatory mediators that create a pro-tumor environment, promoting their survival and metastasis.

NK cells and T cells are crucial in post-surgical immunosurveillance [46]. NK cells are capable of destroying cancer cells without prior sensitization, while Tc cells and Th cells coordinate the immune response against tumor cells. However, surgery significantly reduces the levels of circulating NK and T cells, mainly through the activation of the programmed death-1 (PD-1) and programmed death–ligand 1 (PD-L1) pathway [47]. Cytokine imbalances further exacerbate immune suppression, increasing anti-inflammatory cytokines like IL-10 while reducing pro-inflammatory cytokines such as IFN-γ, thereby shifting the immune response in favor of tumor survival [48].

Treg cells, which are known for their immunosuppressive role, also increase after surgery, promoting a tolerant environment that allows cancer cells to thrive [49]. Elevated Treg levels have been associated with poor prognosis lung cancer and other malignancies [50,51,52]. Furthermore, myeloid-derived suppressor cells (MDSCs), another immunosuppressive cell type, increase after surgery. The recruitment of MDSCs is facilitated by a reduction in chemokine ligand 4 (CXCL4), which is known to inhibit MDSC activity [53]. Elevated MDSC levels have been linked to cancer recurrence and a poor prognosis [54,55,56], as these cells promote tumor progression through angiogenesis and immune suppression [57]. In lung cancer patients, the increased presence of MDSCs after surgery supports angiogenesis and facilitates tumor growth [58].

## 4. Effect of Thoracic Anesthesia on Lung Cancer Recurrence

Given the potential impact of perioperative changes on tumor growth and survival, optimizing anesthetic management to mitigate these effects is essential for improving patient outcomes. In this section, we review commonly used anesthetic agents and techniques in lung cancer resection, focusing on their influence on stress responses, inflammation, and immune function, as well as their potential effects on cancer recurrence and metastasis. To provide a comprehensive overview of current evidence regarding anesthetic agents and techniques used in lung cancer surgeries, we have summarized the major findings from clinical studies in Table 1.

### 4.1. General vs. Regional Anesthesia

Anesthetic techniques may influence cancer outcomes by modulating the immune system and the body’s stress response during surgery, both of which are associated with tumor progression. Regional anesthesia (RA), such as neuraxial and peripheral nerve blocks, has been shown to reduce surgical stress by attenuating the neuroendocrine response, thus preserving immune function [59,60,61]. Preclinical studies suggest that RA may reduce circulating levels of cortisol and catecholamines, potentially limiting tumor cell invasion and metastasis by reducing EMT and maintaining NK cell activity [62,63]. In clinical practice, RA is hypothesized to decrease recurrence risk by modulating the balance between Th1 and Th2 immune responses, thereby enhancing the body’s ability to eliminate residual cancer cells [64]. Additionally, RA may have direct inhibitory effects on cancer cells [65,66] while reducing the need for volatile anesthetics and opioids, both of which are associated with immunosuppression [67,68].

Despite the theoretical advantages, clinical trials have not consistently shown a significant reduction in cancer recurrence or improved survival with RA compared to general anesthesia (GA) alone. A randomized controlled trial (RCT) involving 400 patients undergoing video-assisted thoracoscopic surgery (VATS) for lung cancer compared the use of combined epidural–GA with GA alone [69]. After a median follow-up of 32 months, no significant differences were found between the two groups in terms of recurrence-free survival (RFS), cancer-specific survival, or overall survival (OS) between the two groups. Hazard ratios were 0.90 for RFS (95% CI: 0.60–1.35, *p* = 0.068), 1.08 for cancer-specific survival (95% CI: 0.61–1.91, *p* = 0.802), and 1.12 for OS (95% CI: 0.64–1.96, *p* = 0.697). Similar findings have been reported in other trials assessing RA’s impact on oncologic outcomes [70,71].

One explanation for these mixed results may lie in the complexity of the TME and the variable biological behavior of different cancers. While RA reduces stress hormone levels and preserves immune function, these effects may not be sufficient to counteract the multifactorial nature of tumor recurrence and metastasis. Additionally, the concentration of local anesthetics at micro-metastatic niches may not be high enough to exert a robust anti-tumor effect [72,73].

In summary, although RA offers potential physiological benefits, including reduced stress response and opioid-sparing effects, current clinical evidence does not consistently demonstrate a significant impact on long-term cancer outcomes when compared to GA alone.

### 4.2. Volatile vs. Total Intravenous Anesthetics (Propofol)

Volatile anesthetics, such as isoflurane and sevoflurane, have been extensively studied for their potential impact on cancer progression. Inhalation anesthetics may promote metastasis by activating the hypothalamic–pituitary–adrenal axis and sympathetic nervous system, leading to the release of neuroendocrine mediators such as cortisol and catecholamine [61,74]. These agents suppress immune responses by reducing NK cell activity and increasing the release of immunosuppressive cytokines [75,76,77]. Additionally, volatile anesthetics induce T lymphocyte apoptosis and increase the expression of HIF-1, which is associated with cancer cell proliferation and metastasis via the Akt/mTOR and VEGF pathways [78,79,80]. Studies in NSCLC have demonstrated that isoflurane concentrations of 1–3% enhance both cancer cell proliferation and invasion [78], although other studies suggest that sevoflurane may inhibit invasion by downregulating MMPs and HIF-1α [81,82,83]. This duality highlights the complexity of volatile anesthetics’ effects, which may vary based on the specific cancer cell type and experimental conditions.

In contrast, propofol, a commonly used intravenous anesthetic, has demonstrated anti-tumor properties in both preclinical and clinical studies. Preclinical studies indicate that propofol inhibits tumor cell viability, migration, and invasion by modulating molecular pathways such as STAT3/HOTAIR and by reducing the expression of critical factors like Slug and HIF-1α [79,84,85,86,87,88]. Additionally, propofol promotes apoptosis in lung cancer cells by activating p53 and suppressing ERK signaling, both of which are key regulators of cell survival and metastasis [89]. Propofol also downregulates oncogenes such as neuroepithelial cell-transforming gene 1 and sex-determining region Y box (SOX)4 [86,90,91]. Furthermore, propofol inhibits EMT markers, including N-cadherin and MMPs, reducing the potential for metastasis [92,93,94,95]. Its immune-modulating effects, such as enhanced NK cell activity and reduced levels of pro-inflammatory cytokines like IL-6 and TNF-α, may further contribute to its anti-cancer properties [96,97,98].

Clinical studies have also shown promising results for propofol-based total intravenous anesthesia (TIVA) in cancer surgery [99,100,101,102]. Several retrospective analyses have reported better OS in patients undergoing cancer surgery with propofol compared to volatile anesthetics [103,104,105]. Recent meta-analysis studies found that propofol-based TIVA was associated with improved OS and RFS compared to volatile agents [106,107]. However, not all studies support these findings. Some retrospective studies have reported no significant differences in RFS or OS between TIVA and volatile anesthetics, including in lung cancer cases [108,109]. Other studies have similarly produced mixed results, indicating that propofol may offer some oncological advantages, but the evidence remains inconclusive [110,111].

In summary, while propofol appears to exert anti-tumor effects through immune modulation and the direct inhibition of cancer cell activity, volatile anesthetics may promote tumor progression in certain contexts. However, the available data from both preclinical and clinical studies remain inconclusive, and further research is required to establish a definitive link between anesthetic type and long-term cancer outcomes.

### 4.3. Opioid Agents

Opioids, widely used for perioperative analgesia in cancer surgeries, have raised concerns about their potential role in cancer progression. Laboratory studies indicate opioids can modulate immune function, often leading to immunosuppression [112,113]. Morphine and fentanyl, for instance, reduce NK cell activity, promote lymphocyte apoptosis, and inhibit T cell proliferation [114,115,116]. However, different opioids may have varying immunomodulatory effects. While morphine has been shown to promote tumor growth by enhancing angiogenesis and suppressing immune responses [117], oxycodone has been found to have minimal impact on immune function [118]. Conversely, tramadol may possess immune-stimulating properties, potentially reducing the risk of metastasis [119].

Opioids can directly influence tumor growth by activating transcription factors and promoting angiogenesis through the activation of VEGF receptors [120,121]. These agents also affect cell proliferation through Akt and ERK signaling, while higher doses can induce tumor cell death through NF-κB inhibition and p53 stabilization [122,123].

Additionally, opioids have been linked to enhanced angiogenesis and tumor growth, primarily through the activation of mu-opioid receptors (MOR) in cancer cells [114,124,125]. Preclinical models of NSCLC have demonstrated that MOR activation promotes tumor growth pathways such as Akt/mTOR and VEGF signaling [126,127,128,129]. At the same time, opioid antagonists like methylnaltrexone have shown potential in reducing tumor growth and metastasis [130,131]. The overexpression of MOR in cancer cells is associated with poorer outcomes, including higher rates of recurrence and metastasis, particularly in cancers such as prostate and NSCLC [126,132]. A continuous infusion of methylnaltrexone has been shown to decrease primary tumor growth and lung metastasis [133], suggesting the potential of MOR antagonism as a therapeutic strategy in limiting opioid-driven tumor progression.

The clinical evidence regarding opioid use in cancer patients remains mixed. Some studies suggest that fentanyl administered during or immediately after surgery is associated with poorer OS and RFS in NSCLC [134,135]. However, other studies report no significant impact of perioperative opioid use on long-term oncologic outcomes in NSCLC [136]. Conflicting data also exist for other cancer types, such as colorectal cancer and esophageal cancer [137,138].

Despite the potential cancer-promoting effects of opioids, poorly managed pain may also contribute to tumor progression by increasing sympathetic and adrenal activity, which elevates catecholamine and glucocorticoid levels and suppresses immune function. A retrospective study has linked poorly controlled pain or higher opioid needs to worse survival outcomes in advanced NSCLC patients [139]. Therefore, balancing effective pain management with minimizing opioid use is crucial in determining their impact on cancer recurrence.

### 4.4. Non-Opioid Agents

#### 4.4.1. Nonsteroidal Anti-Inflammatory Drugs (NSAIDs)

NSAIDs exhibit anticancer effects primarily by reducing inflammation and inhibiting PGE2 synthesis [140,141,142]. By inhibiting cyclooxygenase (COX) enzymes, NSAIDs reduce PGE2 levels, suppressing tumor-promoting pathways and enhancing immune responses, particularly Tc cell and NK cell activity [143]. In vitro studies demonstrate that NSAIDs like aspirin and celecoxib reduce cancer cell viability, migration, and proliferation through both COX-dependent and COX-independent mechanisms [144,145,146]. Animal models further show that NSAIDs downregulate oncogenes like SOX2 and reduce VEGF expression, inhibiting tumor growth and metastasis [147].

Clinical studies regarding NSAIDs’ impact on cancer recurrence have yielded mixed results [148,149,150,151]. Regular NSAID use, especially aspirin, has been associated with reduced cancer incidence and improved RFS in some retrospective studies, including NSCLC [152,153]. However, other studies found no significant survival benefits with perioperative NSAID use alone [154,155]. A review of 16 studies concluded that the perioperative effects of NSAIDs on reducing cancer recurrence remain inconclusive [156].

#### 4.4.2. Dexmedetomidine

Dexmedetomidine, a selective α2-adrenoceptor agonist, has demonstrated both pro-tumor and anti-tumor effects depending on the context [157,158]. It interacts with α2 adrenoceptors on both immune and tumor cells, potentially influencing immune regulation and tumor progression.

Preclinical studies suggest that dexmedetomidine may promote cancer cell survival through the upregulation of HIF-1α, enhance metastasis via MMPs, and stimulate angiogenesis by increasing VEGF expression [159,160,161]. In contrast, dexmedetomidine infusion has been shown to increase NK cells, B cells, and CD4+ T cells while improving the CD4+/CD8+ and Th1/Th2 ratios [158]. In animal models, dexmedetomidine has been associated with increased metastasis in cancers such as lung, liver, and colon, particularly through MMP expression and the induction of MDSCs [162,163,164,165]. However, other studies show that dexmedetomidine may reduce metastasis by upregulating miR-143-3p and downregulating EGFR expression [166].

A retrospective study of NSCLC patients reported worse OS with intraoperative dexmedetomidine use, although RFS was not significantly affected [167]. These findings still require confirmation through further clinical trials.

#### 4.4.3. Thiopental

Thiopental, a barbiturate that acts on the GABA-A receptor, has demonstrated immunosuppressive effects in preclinical studies. It suppresses NK cell and neutrophil activity while protecting T lymphocytes from apoptosis [168,169]. This immunosuppression, primarily due to the inhibition of the NF-κB pathway, may contribute to cancer cell survival and metastasis, particularly in lung cancer [77,170]. However, clinical studies have not yet established a definitive link between perioperative thiopental use and oncologic outcomes.

#### 4.4.4. Ketamine

Ketamine, an NMDA receptor antagonist, is widely used for its anesthetic and analgesic properties. Preclinical studies suggest that ketamine may reduce cancer cell proliferation and migration by lowering intracellular calcium levels and inhibiting HIF-1α, p-AKT, and p-ERK expression, thereby reducing VEGF levels [171,172]. Additionally, ketamine decreases pro-inflammatory cytokines, such as IL-6 and TNF-α, which may further inhibit tumor growth [173]. However, ketamine also suppresses NK cell activity, induces lymphocyte apoptosis, and inhibits dendritic cell maturation, which may promote metastasis [77,174,175,176].

In lung adenocarcinoma models, ketamine has been shown to promote apoptosis and inhibit cell growth through CD69 expression [177]. However, some studies suggest an increased risk of metastasis due to reduced NK cell activity [77,174]. Clinical evidence regarding ketamine’s overall impact on cancer outcomes remains limited and inconclusive [178,179].

### 4.5. Local Anesthetics

Local anesthetics (LAs), commonly used for intraoperative anesthesia and postoperative analgesia, block neural transmission by inhibiting voltage-gated sodium channels (VGSCs) [180]. Recent studies suggest that LAs may also have direct anti-tumor effects by modulating cancer cell behavior [181,182]. By reducing the surgical stress response, LAs may help mitigate immunosuppression and preserve the immune system’s ability to eliminate cancer cells. Additionally, LAs reduce the need for opioids and volatile anesthetics, both of which may negatively impact cancer recurrence. Recent evidence suggests that amide LAs may directly inhibit cancer cell growth.

Laboratory studies have shown that LAs, particularly amide types such as lidocaine, can inhibit cancer cell viability, migration, and proliferation in vitro [183,184]. Lidocaine has been shown to reduce lung cancer proliferation by upregulating miR-539, which blocks EGFR signaling [185]. Lidocaine also exhibits anti-inflammatory properties, reducing pro-inflammatory cytokines such as IL-1β, IL-6, and TNF-α, which may help prevent perioperative immunosuppression [186,187]. Additionally, it preserves NK cell function and lymphocyte proliferation, supporting the immune system’s role in targeting cancer cells [188,189,190].

LAs may also reduce metastasis by inhibiting VGSC activity, which is crucial for tumor cell invasion and metastasis formation. Preclinical studies suggest that LAs block the formation of invadopodia, structures that help cancer cells degrade the extracellular matrix and invade surrounding tissues [191,192]. Lidocaine reduces lung metastasis by decreasing MMP-2 levels in murine breast cancer models, limiting tumor cell invasion [193,194]. Both lidocaine and ropivacaine further inhibit cancer cell migration and invasion by blocking TNF-α-induced Src phosphorylation and reducing ICAM-1 expression, which are essential for cellular adhesion in lung cancer cells [195,196]. Furthermore, lidocaine and ropivacaine have demonstrated anti-angiogenic effects by inhibiting VEGF-induced tumor growth and promoting apoptosis in tumor-associated endothelial cells [197,198].

Despite promising preclinical data, clinical evidence on the impact of LAs on cancer recurrence remains mixed. Some retrospective studies have suggested that regional anesthesia, which reduces opioid and volatile anesthetic use, may improve OS in cancer patients [199,200,201]. However, more recent studies, including a Cochrane review, concluded that the evidence supporting the benefit of local anesthetics on cancer recurrence remains inadequate, with conflicting results from various retrospective studies [202,203,204]. Although some clinical studies have shown potential benefits, such as improved survival in patients with pancreatic cancer receiving intravenous lidocaine [205], prospective trials are needed to clarify these findings across various cancer types.

### 4.6. Others

#### 4.6.1. Hypothermia

Perioperative hypothermia can suppress immune function by reducing NK cell activity and disrupting the Th1/Th2 cytokine balance, both of which promote cancer metastasis [206,207]. Retrospective studies show mixed results, with some reporting worse cancer outcomes [208,209], while others find no significant impact on recurrence or survival [210].

#### 4.6.2. Transfusions

Perioperative blood transfusions, often necessary in cancer surgeries, have been linked to immunosuppressive effects that may contribute to cancer recurrence [211,212]. Transfusions can impair macrophage function and shift the immune balance toward a pro-tumor Th2 profile. Retrospective studies associate allogeneic transfusions with poorer OS and disease-free survival in several cancer types, including gastric, bladder, and lung cancers [213,214,215,216]. However, the exact relationship between transfusions and cancer prognosis remains unclear, and more research is needed to understand the underlying mechanisms.

#### 4.6.3. β-Blockers

β-blockers, commonly used as antihypertensive agents, have shown potential anticancer effects by reducing catecholamine-mediated tumor progression [217,218]. In vitro studies suggest β-blockers may exert anti-metastatic effects by reducing inflammation and inhibiting pro-tumor Treg cell activity [219,220]. Retrospective studies in patients with ovarian, breast, and other cancers have indicated improved survival with perioperative β-blocker use [221,222]. Meta-analyses have shown similar trends, although results vary depending on factors such as administration time, cancer stage, and tumor type [223,224,225]. Further studies are needed to confirm the benefits of β-blockers in cancer surgery.

#### 4.6.4. Steroids

Corticosteroids, such as dexamethasone, are frequently used perioperatively for their anti-inflammatory and anti-emetic properties. However, their immunosuppressive effects at higher doses have raised concerns about increased cancer recurrence. Retrospective studies have shown mixed results, with some indicating improved survival in cancers like NSCLC and pancreatic cancer with perioperative dexamethasone use [152,226], while others report worsened outcomes, particularly in colorectal cancer [227,228]. More prospective trials are needed to clarify the long-term impact of corticosteroid use on cancer recurrence and metastasis.

**Table 1:** Summary of clinical studies on anesthetic agents and techniques in lung cancer surgery.

Anesthetic Agents/Techniques	Study Type	Author (Year)	Patients/Studies	Findings	References
EA + GA (vs. GA alone)	RCT	Xu. et al. (2021)	n = 400	No difference in OS and RFS	[69]
EA + GA (vs. GA alone)	RCT	Du. et al. (2021)	n = 1802	No difference in OS and RFS	[70]
TIVA (vs. Volatile)	Meta-analysis	Chang. et al. (2021)	n = 19	Improved OS and RFS	[106]
TIVA (vs. Volatile)	Meta-analysis	Yap. et al. (2019)	n = 10	Improved OS and RFS	[107]
TIVA (vs. Volatile)	Retrospective	Oh. et al. (2018)	n = 943	No difference in OS and RFS	[108]
Opioid	Retrospective	Cata. et al. (2014)	n = 901	Decreased OS and RFS (in stage I)	[134]
Opioid	Retrospective	Maher. et al. (2014)	n = 99	Increased in recurrence rate	[135]
Opioid	Retrospective	Oh. et al. (2017)	n = 1009	No difference in recurrence rate and OS	[136]
NSAIDs	Retrospective	Choi. et al. (2015)	n = 1139	No difference in OS and RFS	[154]
NSAIDs	Retrospective	Lee. et al. (2016)	n = 1637	No difference in OS and RFS	[155]
Dexmedetomidine	Retrospective	Cata. et al. (2017)	n = 1404	Decreased OS and no difference in RFS	[167]

EA: epidural anesthesia, GA: general anesthesia, RCT: randomized controlled trial, TIVA: total intravenous anesthesia, OS: overall survival, RFS: recurrence-free survival, NSAIDs: non-steroidal anti-inflammatory drugs.

## 5. Current Large-Scale Studies and Proposed New Research Directions

Recent clinical trials have sought to elucidate the relationship between anesthetic techniques and cancer recurrence rates in surgical patients, with a particular focus on the effects of volatile anesthetics and TIVA (Table 2). The VAPOR-C trial compares the long-term impact of propofol-based TIVA with volatile anesthesia on RFS in patients with lung and colorectal cancers, aiming to determine whether TIVA provides superior oncologic outcomes. Preliminary results suggest TIVA may have a favorable impact, though comprehensive results are awaited. Similarly, the GA-CARES trial examines various anesthetic agents across multiple cancer types, including lung cancer, to assess their influence on OS and recurrence rates. The GAS-TIVA trial focuses on NSCLC, comparing the recurrence rates between propofol-based TIVA and volatile agents. These studies will provide critical insights into optimizing anesthetic strategies for improved oncologic outcomes.

Beyond these large-scale studies, new research should investigate how anesthetic agents modulate molecular mechanisms such as ferroptosis and autophagy, which are crucial in cancer cell survival and death [229,230]. Ferroptosis is a form of regulated cell death characterized by lipid peroxidation driven by iron-dependent processes. It contrasts with apoptosis and necrosis by involving unique mechanisms such as glutathione peroxidase 4 (GPX4) inhibition, leading to cellular damage and death. Autophagy, on the other hand, plays a dual role by promoting cell survival under stress but can also trigger ferroptosis through processes like ferritinophagy, which releases free iron and generates reactive oxygen species. These mechanisms represent promising targets for therapeutic strategies, suggesting that anesthetic techniques impacting oxidative stress and autophagic activity could influence cancer outcomes. Anesthetics like propofol and dexmedetomidine are known to interact with these mechanisms; propofol can modulate oxidative stress and autophagic processes, while dexmedetomidine may inhibit ferroptosis by enhancing GPX4 expression. Understanding these interactions could reveal how perioperative anesthetic choices impact cancer cell viability and long-term recurrence, opening new therapeutic strategies that combine anesthetic management with targeted interventions.

## 6. Conclusions

While increasing evidence suggests that anesthetic techniques and perioperative management may influence cancer recurrence and metastasis, much of the current data come from preclinical or retrospective studies with conflicting results. Certain anesthetic agents, such as propofol, have shown promising anti-tumor effects, whereas others, such as volatile anesthetics and opioids, have been linked to tumor-promoting mechanisms. However, these findings are not entirely consistent, likely due to the complex interactions between tumor biology, surgical techniques, and patient-specific factors such as immune status, comorbidities, and genetics. This complexity makes it challenging to isolate the effects of individual agents or techniques on cancer outcomes.

In addition to anesthetic agents, future studies should focus on other perioperative factors such as pain management, blood transfusions, and perioperative hypothermia, which may significantly affect cancer prognosis. Understanding the influence of these variables is crucial to developing comprehensive perioperative strategies aimed at reducing metastasis risk and improving survival.

Effective anesthetic management in cancer surgery requires balancing immediate perioperative needs with long-term oncologic outcomes. Personalized approaches, considering each patient’s risk profile—including immune status and comorbidities—are essential. Multidisciplinary collaboration between anesthesiologists, surgeons, and oncologists is key to ensuring that perioperative care effectively supports oncologic considerations.

## Figures and Tables

**Table 2:** Ongoing prospective randomized clinical trials on anesthetic management and lung cancer recurrence.

Trial Registry Number	Study Title	Study Design	Interventions	Primary Outcome	Estimated Completion
NCT03034096	General Anesthetics in Cancer Resection Surgery (GA-CARES trial)	All cancer type (n = 2000)	Propofol-based TIVA vs. Sevoflurane, Isoflurane, Desflurane	All-cause mortality	December 2025
NCT04316013	Volatile Anaesthesia and Perioperative Outcomes Related to Cancer (VAPOR-C trial)	Non-small cell lung cancer, colorectal cancer (n = 3500)	Propofol-based TIVA vs. Sevoflurane	Disease-free survival	June 2028
NCT06330038	Recurrence Free Survival After Curative Resection of Non-small Cell Lung Cancer Between Inhalational Gas Anesthesia and Propofol-based Total IntraVenous Anesthesia (GAS-TIVA trial)	Non-small cell lung cancer (n = 5384)	Propofol-based TIVA vs. Sevoflurane, Isoflurane, Desflurane	Recurrence-free survival	December 2028

TIVA: total-intravenous anesthesia.
